# Modeling influence and community in social media data using the digital methods initiative-twitter capture and analysis toolkit (DMI-TCAT) and Gephi

**DOI:** 10.1016/j.mex.2020.101164

**Published:** 2020-11-27

**Authors:** Jacob Groshek, Vincent de Mees, Rob Eschmann

**Affiliations:** a Kansas State University, United States, Roskilde University, Denmark; b AnimatingScience, SlimPlot, The Netherlands; c Boston University, United States

**Keywords:** Network analysis, Data visualization, Algorithmic sorting and filtering

## Abstract

The methods summarized in this video tutorial series are based on the open source Digital Methods Initiative – Twitter Capture and Analysis Toolkit (DMI-TCAT) that allows media researchers to collect tweets off the STREAM API (application programming interface) on an ongoing basis. With DMI - TCAT and the open source data visualization software Gephi, social data in the millions of units is quickly and easily sorted by algorithms to find users or items of importance on Twitter, such as in the Fig. 1 below.

While these figures and the data gathered though the DMI-TCAT do not provide full firehose access to all historical tweets, they do provide a generally representative sample of tweets that is relatively proportional to the total volume of tweets being posted at any given time (Gerlitz & Rieder, 2013; Groshek & Tandoc, 2016). For more details on the DMI-TCAT and its operation, we encourage readers to visit its github page (https://github.com/digitalmethodsinitiative/dmi-tcat) and note that this cloud-based analytics program is free and customizable.

The specific techniques covered in the methodology reported here in text and expanded upon in the video tutorial series include how to:

• Model influence users by sizing nodes with the betweenness centrality algorithm;

• Identify community groups by adding color using the modularity algorithm;

• Spatialize networks through applying the openord algorithm;

• Make social network graphs dynamic and interactive online.

## Graphical abstract

**Figure:**
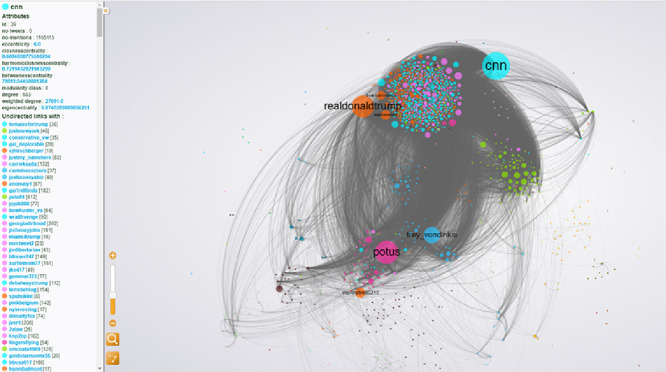

Specifications Table

**Table:** 

Subject Area	Computer Science
More specific subject area	*Sociology, media and communication, computational social science*
Method name	*Creating interactive social network analysis graphs*
Name and reference of original method	Eschmann, R., Groshek, J., Li, S., Toraif, N., & Thompson, J. G. [2]. Bigger than sports: Identity politics, Colin Kaepernick, and concession making in# BoycottNike. *Computers in Human Behavior*, 106583. https://doi.org/10.1016/j.chb.2020.106583 Borra, E., & Rieder, B. [1]. Programmed method: Developing a toolset for capturing and analyzing tweets. *Asian Journal of Information Management, 66(3), 262–278.* doi:10.1108/AJIM-09-2013-0094
Resource availability	https://github.com/digitalmethodsinitiative/dmi-tcat https://gephi.org/ https://filezilla-project.org/

## Method details

Social media intersects with nearly every aspect of personal and public life in contemporary society. Still, social media platforms are not discipline specific and can cover a wide range of methodological approaches. Data-driven research that relies on computational analyses of social media data has grown tremendously in volume and rigor, and this is why the outlined methodology fills a vital, topically agnostic need. The method described here was used to study tweets surrounding former professional football player Colin Kaepernick's decision to kneel as a form of protest against police violence against Black people, and what user behaviors and characteristics were associated with users who were anti-Kaepernick making concessions, or signaling a willingness to accept a different point of view [2].

**Fig. 1:**
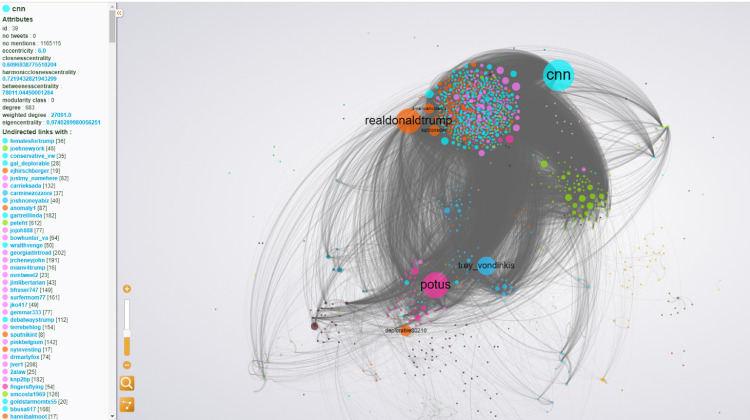
Network graph of 2495,827 users in 14,309,930 tweets about fakenews (01/03/17 - 05/07/2018) summarized into 1500 nodes and 56,505 undirected edges. Spatialization: OpenOrd (w/Noverlap); Size: Weighted Degree; Color: Modularity.

The Digital Methods Initiative – Twitter Capture and Analysis Toolkit (DMI-TCAT) allows researchers to collect tweets off the STREAM API (the so-called “gardenhose” access to Twitter) and then processes that data for network analysis and visualization in Gephi, R, Excel, SPSS, or other similar applications. Importantly, with the DMI-TCAT software, social media data in the millions of units can be preprocessed to effectively use and sort by algorithms to find users, content, patterns, or items of importance on Twitter. While the DMI-TCAT does not collect every tweet posted, this open source software nonetheless captures representative samples of public tweets, and it is free and customizable for anyone wishing to use it.

Social network analysis (SNA) is the process of investigating social structures through the use of network and graph theories. It characterizes networked structures in terms of nodes (individual actors, people, or things within the network) and the ties, edges, or links (relationships or interactions) that connect them. These networks are often visualized through network graphs in which nodes are represented as points and ties are represented as lines. Every network graph depends on nodes and edges, and how that can be modeled, interpreted, and understood.

One especially important feature of SNA is differentiating between directed and undirected edges. While there are different topics to which this concept can be applies, in the case of Twitter data, directed edges point to a specific node such as @mentioning a user or messaging a user, where actions are not technological required to be *reciprocal*. Undirected edges, on the other hand, are models where all edges are bidirectional and connected together, such as co-occurring hashtags or word clouds, where reciprocity is built in as a function of the text being analyzed.

By using the DMI-TCAT as a portal into social media data, it is possible to engage three key elements that can help to explore and explain trends and patterns within big social media data using the free and open source data visualization application Gephi:

1. Color nodes to identify communities
2. Size nodes to identify users (or content) of prominence
3. Spatialize graphs to identify network structure

In addition, it is possible to export data into both visual and statistical outputs. Much of this work can not be done in the browser (at least not yet) and these analyses gain insights into many areas related to users and content around certain topics of information flows on Twitter. Each of these processes are briefly summarized here, with graphical and step-by-step video instructions provided in the accompanying links.

As an example of user analysis, before beginning the analytical process in Gephi, it is important to load the topic of interest into the DMI-TCAT, and then select ‘Social graph by mentions’ → launch. At this point, you can select 1500 top users (this is an approximate starting point, but adjustable to the size of the corpus) and then right-click → ‘save link as’ to local folder or directory. Once completed, to complete the three-step process mentioned above, follow these steps, which are also available in video tutorial form here https://vimeo.com/showcase/4599762

1. Color nodes to identify communities

• Open downloaded file in Gephi
• File → Open
• Select the appropriate ‘directed’ vs. ‘undirected’ vs. ‘mixed’ graph option
• Modularity → Run
• Partition → Palette
• Apply
• Nodes should now have colors applied by community

2. Size nodes to identify users (or content) of prominence in Gephi

• Network Diameter→ Run
• Ranking→ Betweenness Centrality
• Apply
• Nodes should now have size applied by Betweenness Centrality

3. Spatialize graphs to identify network structure

• OpenOrd→ Run
• Noverlap→ Run (alternate ‘Label Adjust’ → Run)
• Nodes should now be spatialized by the OpenOrd force-directed algorithm

Once these steps are completed, it is possible to export into visual and statistical output. Here, it is worthwhile to note that betweenness centrality is a measure of the extent to which a node is connected to other nodes that are not connected to each other. While it is a useful measure of the degree to which a node serves as a bridge or gatekeeper to otherwise unconnected users, there are many other measures of centrality that can be applied within Gephi or other platforms. Similarly, there are many other ways to color and spatialize nodes from DMI-TCAT output, this is just one example.

*Please see* https://vimeo.com/showcase/4599762 *for complete series of online video tutorials.*

*A sample interactive graph that corresponds to the online video tutorials is available here* http://www.gorilladragon.org/fake_news/

*Sample data for the interactive graph that corresponds to the online video tutorials is available at the same link (*http://www.gorilladragon.org/fake_news/*). In order to access that data, right click and ‘Save Data As’ on the ‘Download data here’ link.*

## Declaration of Competing Interest

The authors declare that they have no known competing financial interests or personal relationships that could have appeared to influence the work reported in this paper.
